# Applications of Artificial Intelligence in Out-of-Hospital Cardiac Arrest: A Systematic Review

**DOI:** 10.7759/cureus.82320

**Published:** 2025-04-15

**Authors:** Doju Cheriachan, Heet N Desai, Leslie Sangurima, Maujid Masood Malik, Nency Ganatra, Rosemary Siby, Sanjay Kumar, Sara Khan, Srilakshmi K Jayaprakasan, Pousette Hamid

**Affiliations:** 1 Internal Medicine, California Institute of Behavioral Neurosciences and Psychology, Fairfield, USA; 2 Biomedical Sciences, King Faisal University, Hafuf, SAU; 3 Internal Medicine, Bahria University Medical and Dental College, Pakistan Navy Ship (PNS) Shifa Hospital, Karachi, PAK; 4 Paediatrics, Dr. B. R. Ambedkar Medical College and Hospital, Bengaluru, IND; 5 Neurology, California Institute of Behavioral Neurosciences and Psychology, Fairfield, USA

**Keywords:** ai protocols for healthcare, artificial intelligence in health care, cardiac arrest outcome, cardiac resuscitation, emergency response, management of cardiac arrest, out-of-hospital cardiac arrest

## Abstract

Artificial intelligence (AI) refers to a computer system capable of performing complex tasks that require human skills. AI is increasingly being utilized in the healthcare sector; therefore, the aim of this review was to explore the role of AI in enhancing immediate response and successful management of cardiac arrest by improving the recognition of cardiac arrest from emergency calls in an out-of-hospital setting. The preferred design for this study was a literature review. To get the relevant articles, an in-depth literature search for primary and secondary studies was carried out over various databases, namely ProQuest, CINHAL, PUBMED, and Google Scholar. The results revealed that in five out of the 12 studies, there was a total of 98,922 participants. Three were reviews of past studies, and one involved the examination of a number of emergency calls, but the number of participants was not specified, while the other examined AI and self-care. A thematic analysis of the 10 articles was performed and four dominant themes were identified, namely AI improves self-care; AI improves clinical outcomes with a positive predictive value of (33.0%, p < 0.001); improved decision making with an accuracy of 0.908, which increases the survival rate with an accuracy of 0.896; and the prediction of cardiac arrest with an accuracy of 0.8 in predicting cardiovascular risks including cardiac arrest. Hence, it is concluded that the integration of AI facilitates the early prediction of potential cases of cardiac arrest in out-of-hospital settings. Early detection is associated with improved decision-making regarding the next action steps to take. AI enhances self-care, whereby through virtual doctors and online applications, patients can take proactive measures when they are susceptible to a heart attack. As a result, the integration of AI significantly improves patient outcomes.

## Introduction and background

Recent advancements in technological knowledge are revolutionizing the healthcare sector. The development of artificial intelligence (AI) has significantly improved decision-making, enabling care providers to make accurate and timely healthcare decisions that boost patient health outcomes. AI is defined as a computer system capable of performing complex tasks that require human skills [1]. Based on the given definition, AI encompasses the computer system’s ability or capacity to gather and process data to gain in-depth foresight that it uses to carry out complex but specific tasks. In line with the above, AI can think, analyze, perceive, and initiate action by imitating the intelligent behavior of human beings to complete tasks, such as the diagnosis process by a doctor. The management of cardiac arrest is one of the areas that have benefited from the implementation of AI [2]. The application of AI is key to curbing the rising cases of cardiac arrests. For example, it is estimated that 292,000 adults suffer cardiac arrest in the US annually, which constitutes a 38% increase over the last decade [3]. The statistics above prompt the need for a more accurate cardiac arrest prediction.

The implementation of AI increases the timely response and successful management of cardiac arrest in individuals who are at risk of it. It is commonly known that out-of-hospital cardiac arrest (OHCA) is a serious threat to the lives of those who sustain it; therefore, its early identification is vital for the initiation of cardiopulmonary resuscitation (CPR) to save the lives of patients during emergencies inside the hospital setting [4,5]. However, predicting or identifying patients at risk of cardiac arrest for timely CPR is difficult. Despite this, machine learning (ML) frameworks are known to help in effective screening and clinical decisions, hence, prompting the need for AI assistance. In this regard, care providers currently utilize AI and deep learning (DL), in particular, to facilitate an early prediction of individuals who are susceptible to cardiac arrest [6]. DL constitutes an artificial neural network (ANN), which is an algorithm centered on the interconnections of biological neural networks and high data processing nodes. It also entails convolutional neural networks (CNNs), which are networks capable of extracting high-level features from 2D and 3D classification of data; hence, it is applied in real-time prediction of cardiac arrest with an accuracy of 80% in OHCA [7]. The accuracy is predicted to rise to 90-95%. Therefore, DL boosts timely prediction and response to cardiac arrest in OHCA.

However, there are several gaps in knowledge in the application of AI in addressing cardiac arrest in out-of-hospital settings. First, existing literature focuses more on AI use in the prediction of cardiac arrest risk inside hospital settings; therefore, there is a gap in knowledge on the effectiveness and impact of AI in enhancing immediate response and successful management of cardiac arrest in out-of-hospital settings. Second, there is a gap in knowledge in the use of AI frameworks in the recognition of OHCA from emergency calls. In this regard, the current study aims to explore the role of AI in enhancing immediate response and successful management of cardiac arrest by improving the recognition of cardiac arrest from emergency calls in an out-of-hospital setting. Addressing the gaps above will improve emergency medical services and CPR by enabling a timely and accurate prediction of and appropriate response to cases of OHCA.

Existing studies show that the implementation of AI has a positive impact on the management of OHCA. AI enhances individual self-care, which is an effective pro-active and preventative measure that enhances the clinical outcome of those who are at risk of cardiac arrest in out-of-hospital settings [8]. Here, the AI helps the patient to take appropriate measures to manage or prevent the risk of cardiac arrest on their own. In a tight link to this, the integration of AI is useful in enhancing clinical outcomes. Past studies reveal that when AI is used to care for patients within out-of-hospital settings, there is a significant improvement in the prediction of cardiac arrest, treatment and management of cardiac arrests, and clinical outcomes of patients [9,10]. This is attributable to the ability of AI to enhance adherence to appropriate defibrillation guidelines in case of cardiac arrest, as well as automated external defibrillator delivery features. Besides, the use of AI increases the survival rate of those who sustain cardiac arrest in out-of-hospital settings.

Accurate prediction of an impending or the likelihood of an occurrence of cardiac arrest is a key challenge within out-of-hospital settings. However, existing research studies show that the integration of AI has high efficacy in predicting a case of cardiac arrest. DL algorithms enable caregivers or dispatchers to identify individuals who are at risk of cardiac arrest [11-13]. Besides, ML frameworks enable care providers to determine patients who are likely to experience cardiac arrest from emergency calls [10,14]. Accurate predictions facilitate a timely response and resuscitation that saves the lives of many vulnerable individuals.

## Review

Methods

Design

The cornerstone of our study was a comprehensive literature review, a design we chose for its ability to provide a robust foundation for our research. Following the guidelines of the Preferred Reporting Items for Systematic Reviews and Meta-Analyses (PRISMA), we meticulously identified, selected, and appraised past studies related to the phenomenon under investigation. This method, which involves a thorough research process, was instrumental in our search for both primary and secondary journal articles on the concepts of AI in OHCA, as described below.

Search Strategy

To get the relevant articles, an in-depth literature search for primary and secondary studies was carried out over various databases, namely ProQuest, CINHAL, PUBMED, and Google Scholar. To narrow the research to more specific articles, we used BOOLEAN operators 'AND' and 'OR' to combine several keywords, namely: ‘Artificial Intelligence,’ ‘Cardiac arrest,’ ‘resuscitation,’ ‘management,’ and ‘out-of-hospital.’ The Medical Subject Headings (MeSH) search strategy was also used to enhance the search using the keywords in cases where we utilize different words in describing AI in out-of-hospital prediction of cardiac arrest. This was applied as follows: ‘Artificial Intelligence [MeSH],’ ‘Cardiac arrest [MeSH],’ ‘resuscitation [MeSH],’ ‘management [MeSH],’ ‘Heart Arrest [MeSH],’ ‘emergency call in [MeSH],’ ‘deep learning [MeSH],’ ‘machine learning [MeSH],’ ‘intensive care unit [MeSH],’ ‘respiratory failure [MeSH],’ ‘respiratory failure [MeSH],’ ‘Cardiopulmonary resuscitation [MeSH],’ ‘Out-of-hospital cardiac arrest [MeSH],’ ‘Prediction time [MeSH],’ ‘Dispatch-assisted cardiopulmonary resuscitation [MeSH],’ ‘defibrillators [MeSH],’ and ‘out-of-hospital [MeSH].’

Eligibility Criteria

As a strategy for including only relevant and appropriate journal articles in the study, we used a set of eligibility criteria. Several filters were applied directly during the database search. Filters that were used include language, type of publication (peer-reviewed), full-text, and date of publication, which were applied as follows. One of the eligibility criteria was the use of current publications - articles published within the last five years. In this regard, only articles that were published in the last five years, particularly from 2018 to 2022, were included in the study. The period above was chosen to ensure that only journals with the most recent evidence on the application of AI in OHCA are included and to ensure that current trends in AI use in OHCA are captured. Peer review and full publication availability were other eligibility criteria for selecting articles used in this study. The research focused only on including articles that are peer-reviewed and are available in full text. The use of the English language in the publication of an article was another eligibility criterion that was used in selecting articles for use in this study.

The exclusion of articles published in non-English languages and those that were published more than five years ago is associated with limitations. First, excluding articles that are not published in the English language means the omission of potential high-quality evidence-based practice (EBP) on the application of AI in OHCA from non-English-speaking regions. Second, it introduces language bias since the evidence that will be presented in this paper will not be applicable to non-English-speaking regions due to the language barrier. Third, the evidence collected from journal articles published in the English language only will not be generalizable to healthcare settings in non-English-speaking audiences. Besides, the exclusion of articles published more than five years ago also means the omission of valuable scientific evidence on the EBP practices that inform the current AI application in OHCA.

Quality Assessment

In order to establish the rigor of this systematic review, the research subjected the sources that were included in the study to a thorough quality assessment. In line with this, the three investigators (DC, HD, LS) used the eight-question Joanna Briggs Institute Tool for all the studies that were included. The risk of bias in every study outcome was examined, and every journal article was given a score below 4, indicating low quality and high risk for bias. On the other hand, a score of >4 was assigned to articles that have a high quality and low risk of bias, as summarized in Table 1.

**Table 1:** Characteristics of included studies RCT: randomized controlled trial; AI: artificial intelligence

Author	Country	Design	Setting	Sample	Measure	Risk of Bias	Quality	Outcome
Jerkeman et al., 2022 [8]	Sweden	Cross-sectional	Hospital	106,296	Survival rates	Low	High	AI increase of survival rates
Kim et al., 2019 [9]	South Korea	Cross-sectional	Hospital	29,181	Cardiac arrest prediction	Low	High	AI accurately predicts cardiac arrest
Alamgir et al., 2021 [10]	Not specified	Systematic review	Online databases (Scopus, Science Direct, Embase, the Institute of Electrical and Electronics Engineers, and Google Scholar)	41	Prediction of cardiac arrest	Low	High	AI boosts the accuracy of cardiac arrest occurrence prediction
Blomberg et al., 2019 [11]	Denmark	Quantitative study	Hospital	108,607	Recognition of cardiac arrest	Low	High	AI enhances the recognition of cardiac arrest
Blomberg et al., 2021 [12]	Not available	Systematic review	Online databases (Medline and Embase)	Not available	Access to defibrillators	Moderate	Medium	AI eliminates barriers and increases access to defibrillators
Brooks et al., 2022 [13]	Denmark	RCT	Healthcare facility	5242	Machine learning in recognition of cardiac arrest	Low	High	Machine learning enhances the out-of-hospital recognition of cardiac arrest
Barrett et al., 2019 [14]	North-West Europe	Prevalence study	Not available	Not available	Role of AI in prediction, prevention, personal care	Low	High	AI enhances the prediction, prevention, personal care
Scholz et al., 2022 [15]	Denmark	Cross-sectional	Patient database	9049	Efficacy of speech recognition in predicting stroke	Low	High	Speech recognition enhances the prediction of stroke/cardiac arrest
Johnson et al., 2020 [16]	Europe and Australia	Qualitative	Clinical centers	939	Prediction of cardiac arrest	Low	High	AI boosts the accuracy of cardiac arrest occurrence prediction
Ballinger et al., 2018 [17]	Not available	Systematic review	Online databases (MEDLINE, CINAHL, PubMed and the grey literature)	92 studies	Prediction of cardiac arrest	Low	High	Machine learning enhances prediction of cardiac arrest
Harford et al., 2022 [18]	USA	Cohort study	University	14,011	Prediction rates	Low	High	AI facilitates early prediction of cardiac arrest
Miles et al., 2022 [19]	USA	Cross-sectional	Chicago Fire Department	2398	Efficacy of machine learning in cardiac arrest management	Low	High	Machine learning boosts the workflow and management of cardiac arrest

Results

**Figure 1:**
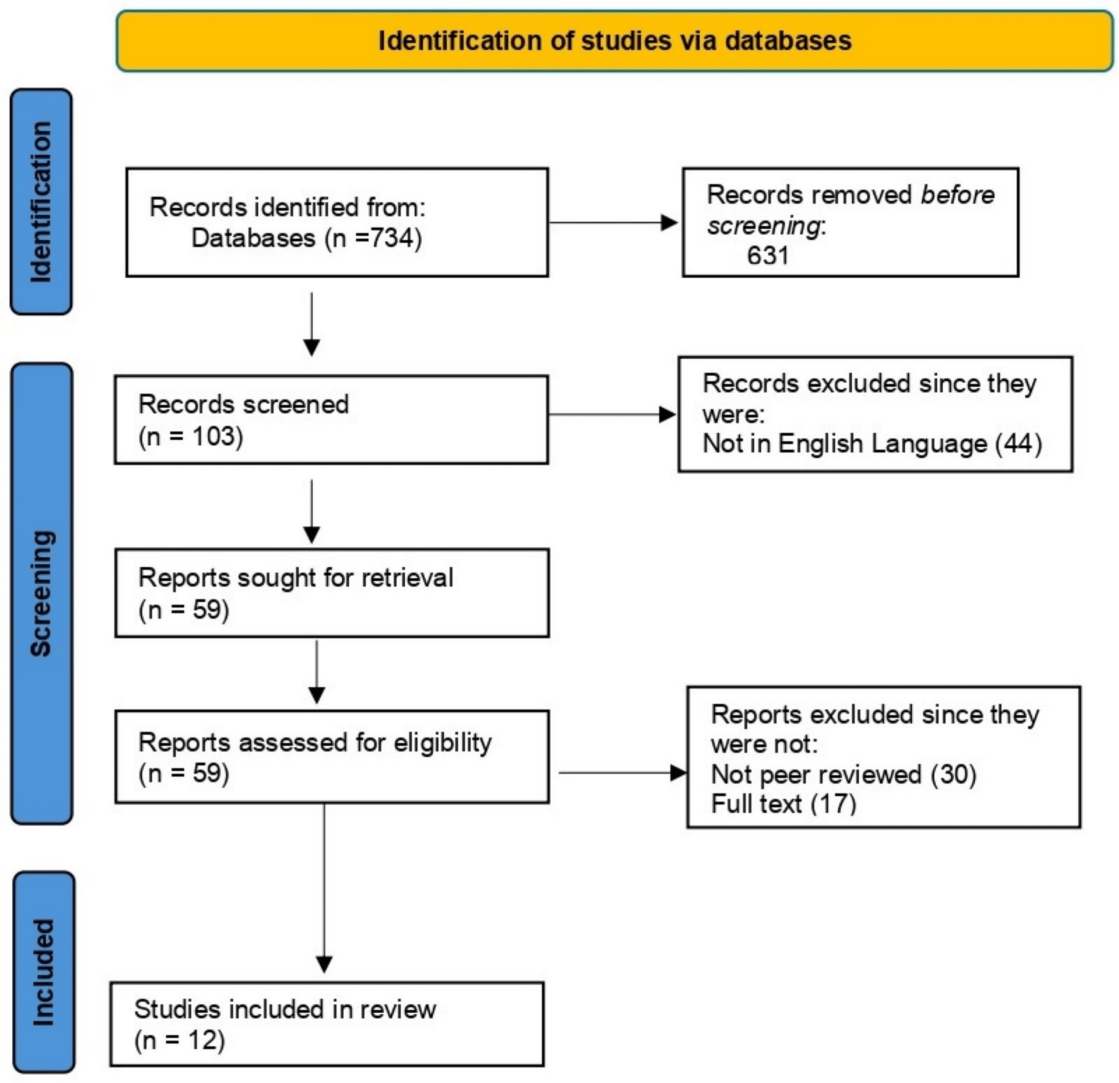
PRISMA flow diagram PRISMA: Preferred Reporting Items for Systematic reviews and Meta-Analyses

The literature search process is illustrated in Figure 1.

Based on the eligibility criteria, the articles identified on the concept of AI in OHCA revealed a total of 734 articles, categorized as follows: AI and cardiac arrest (1990-present) - 734; AI in emergencies (2000-present) - 502; AI in and out of hospitals (2010-present) - 225; AI in out-of-hospital settings (2013-present) - 157. Of these, 123 were published between 2017 and the present; however, the number of articles published in the last five years and retained was 103.

The remaining 103 articles retrieved were further screened for relevance and suitability to be included in the study. Those that were not in the English language and were removed were 44, non-peer reviewed were 30, and those that were not full-text were 17; therefore, only 12 articles were found eligible to be included in the study. In five out of the 12 studies, there was a total of 128,103 participants [8,9,15-19], while two were reviews of past studies [10,12] and one involved the examination of a number of emergency calls, but the number of participants was not specified [11,13], while the other examined AI and self-care [14]. Regarding the areas of focus, the dominant themes were self-care [12], improved clinical outcomes [12,16], improved decision making [19], and the prediction of cardiac arrest [10,13,15,17,18].

Outcomes of studies

Early Prediction of Cardiac Arrest

The results of six studies [9,10,13,15,17,18] revealed that the early prediction of cardiac arrest is one of the applications and benefits of integrating AI in the out-of-hospital setting. In a systematic review of 47 articles, 55% of the studies predicted cardiac arrest, 34% of the studies utilized an AI-based warning system in predicting cardiac arrest, and 11% of studies distinguished patients based on the risk of developing cardiac arrest [10]. ML has a high sensitivity for predicting cardiac arrest in OHCA (85%; p < 0.001) [13]. The use of automatic speech recognition (ASR) software improves stroke prediction and successful treatment by 61.19% [15]. For patients with critical care needs, the application of neural networks (ML) in triaging showed an acuity of 0.85 (0.84-0.88), a median C-statistic of 0.89 (0.86-0.91), and a logistic regression of 0.83 (0.79-0.84) [17]. Another study found that AI has an accuracy of 0.8 in predicting cardiovascular risks, including cardiac arrest [18].

Improved Decision-Making

The findings of a single study among the eligible articles that were included in the study revealed that the application of AI improves decision-making in OHCA. The application of ML enhances treatment-related decision-making with an accuracy of 0.908, which increases the survival rate with an accuracy of 0.896 [19].

Improved Patient Clinical Outcomes

The application of AI is associated with improved patient outcomes in OHCA [8]. From 108,607 emergency calls, ML has a positive predictive value of (33.0%, p < 0.001), which informs appropriate and timely treatment for improved outcomes [11]. Innovative approaches to improving access to public defibrillators increase the use of the devices to more than the current rate of <3% in cardiac arrest episodes, hence contributing to improved patient outcomes in OHCA [12]. Based on a total of 54 patients’ clinical variables used in a recent cohort study, the use of an ANN model demonstrated significantly better performance (p = 0.029), with an area under the curve (AUC) exceeding 0.852 [16].

Improved Self-Care

A study examining the application of AI in OHCA reveals a shift from the initial reactive response to cases of cardiac arrest to a predictive, preventive, and more personal care approach to addressing episodes of cardiac arrest [14]. The above is attributable to AI’s ability to promote therapy monitoring, patient-centered care, patient engagement, and patient-supporting technologies.

Discussion of findings

Early Prediction of Cardiac Arrest

**Figure 2:**
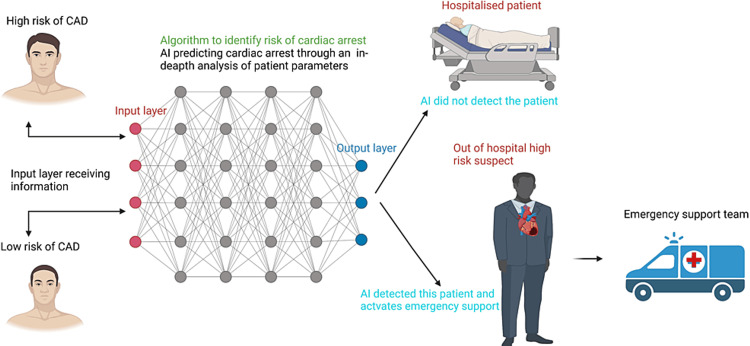
Artificial neural network model predicting out-of-hospital cardiac arrest CAD: coronary artery disease; AI: artificial intelligence Image Credits: Doju Cheriachan

Early prediction of cardiac arrest is one of the benefits of integrating AI in the out-of-hospital setting. In a scoping review that explored the utilization of AI in the prediction of cardiac arrest, it was found that this technology has high efficacy in predicting the likelihood of cardiac arrest in patients within the out-of-hospital setting. AI was found to predict cardiac arrest based on three unique categories using both real-time data collected from patients’ vital signs and existing medical history [10]. First, AI predicts cardiac arrest through an in-depth analysis of patient parameters or variables, such as patients with high risk or no risk of cardiac arrest, those with a history, and those without a history of cardiac arrest. AI tools like heart rate sensors can predict the likelihood of an occurrence of cardiac arrest by measuring patient vital signs such as high cholesterol (0.7441), diabetes (0.8451), sleep apnea (0.8298), and high blood pressure (0.8086) [10,18]. The above vital signs are major risk factors for cardiac arrest, and their presence is used in predicting OHCA. Second, AI uses ML and DL approaches that use algorithms to identify patients who are at a greater risk of cardiac arrest. Finally, AI analyzes the data set of patients who are not in the hospital setting to determine those who are vulnerable to cardiac arrest (Figure 2).

The above findings are supported by other existing studies that found that ML models that are trained to identify cardiac arrest within out-of-hospital settings enhance the recognition and instant responses to cardiac arrest emergency calls [13,15]. In these studies, the authors found that the integration of AI in practice is associated with an increased percentage of cardiac arrest prediction in out-of-hospital settings compared to when standard protocols only are applied. Improved prediction is made possible through the use of ASR software, which heightens OHCA in emergency settings [15]. ML and DL features, in combination with ASR, increase the ability of dispatchers or care providers to predict the occurrence of cardiac arrest when they receive calls from patients or their family members [17].

AI and Improved Decision-Making

**Figure 3:**
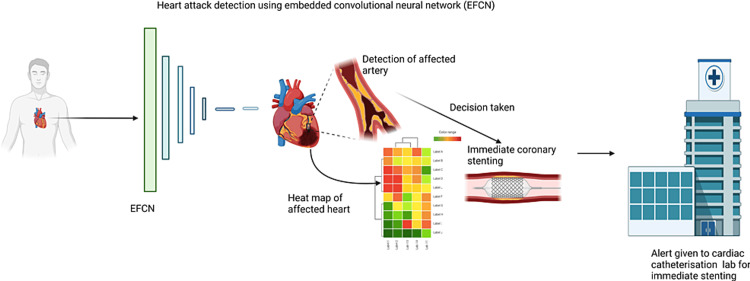
Decision-making with the help of embedded fully convolutional network (EFCN) Image Credits: Doju Cheriachan

The ability to predict the likelihood of cardiac arrest in patients is associated with effective decision-making. ML, in particular, accurately predicts how a combination of various variables influences the patients in out-of-hospital settings, which enhances their ability to make effective response and management decisions [17]. Decision-making in healthcare entails analysis and interpretation of large amounts of data, which can be daunting. However, the application of AI, ML and simulations, in particular, enables healthcare practitioners’ ability to analyze and interpret huge amounts of data consisting of patients’ history, vital signs, risk factors, and other crucial variables promptly and accurately [20]. As a result, care providers can make effective decisions regarding prognosis, diagnosis, treatment, care provider’s availability, and clinical workflow. Using ML models such as embedded fully convolutional networks (EFCN), care providers are assisted in the decision-making process. The EFCN is efficient in performing thorough, in-depth imaging of patients’ cardiovascular systems during coronary angiography. The results provide comprehensive imaging data that is useful in predicting the future occurrence of OHCA. As a result, they can tell the most appropriate action to take, for example, when to perform coronary angioplasty to help restore spontaneous circulation (ROSC) in the patient (Figure 3).

AI and Improved Patient Outcomes

In a tight link to the two themes above, the incorporation of AI comes along with improved outcomes for patients in the out-of-hospital setting. Timely prediction of a possible occurrence of cardiac arrest and the assisted decision-making process improves the quality of service delivery to those who are at risk of cardiac arrest [13]. The occurrence of a cardiac arrest is associated with potential death, especially when the response is delayed for more than 15 minutes [12]. A lack of immediate response is common outside the hospital, especially due to the lack of appropriate knowledge of resuscitation. Additionally, the likelihood of death is possible during transportation to the hospital since emergency medical dispatchers can miss up to 25% of cardiac arrest cases without the assistance of AI [11]. However, the integration of AI has proved effective in fostering the outcome for patients, restoring normal circulation, and preventing death [11,12,16]. In this regard, it suffices to state that the improved outcomes are hugely attributable to the AI’s ability to alert and inform medical dispatchers or emergency care providers of those who are at high risk of cardiac arrest for instant response to save their lives. In this context, those who are at high risk of OHCA are determined through the medical history of cardiac arrest and the vital signs scores as revealed by data from ML. For example, a patient with hypertension and diabetes is at a high risk of OHCA.

Despite the positive role of AI in boosting patient outcomes in OHCA, there are various limitations to using AI in this context. Addressing the impacts of confounding factors is a major challenge for using AI in the OHCA setting. AI tools may fail to predict and identify all the underlying factors causing cardiac arrest and their appropriate treatment or management, which can adversely impact patient outcomes [18]. The effective deployment of new algorithms in OHCA can be challenging, especially when the involved healthcare practitioners are not well-trained and equipped to utilize the algorithms. Subsequently, poor interpretability can lead to a lack of proper cause and effect explanation and reasoning that adversely hamper patient outcomes.

AI and Improved Self-Care in OHCA

Patients who are in hospital emergency departments have a higher survival chance since they are under constant monitoring by care providers and can receive immediate resuscitation when under a heart attack. However, those in the out-of-hospital setting are at a greater risk of death due to the lack of quick response. The inability to take care of oneself is one of the key factors of poor outcomes for patients who are susceptible to cardiac arrest in out-of-hospital settings. However, the emergence of AI has been instrumental in encouraging patient outcomes by advancing self-care. Through AI assistance, patients have become actively involved in the management of this condition by utilizing applications that are accessible online and powered by AI [14]. Even though the study’s central focus was heart failure, the authors examined the application of AI in self-care - monitoring and self-management tasks of cardiac arrhythmia and myocardial infarction, which are precursors of cardiac arrest. An example of an AI-powered self-care application is the atrial fibrillation app. The application has user-friendly features enabling patient education, patient and care-provider real-time monitoring of the patient’s vital signs and patient-care provider engagement [14].

The emergence of self-care complements traditional emergency care by transforming it from reactive to proactive and preventive care; hence, improved mortality rates are attributable to OHCA. Self-care also complements traditional emergency care by eliminating the barrier between care providers and patients since patients are now actively involved in the monitoring and treatment processes, hence improving patient satisfaction. Despite the positive roles of AI applications in patient self-care and self-management efforts, several challenges affect at-risk populations. Digital illiteracy is a notable barrier to the effective use of AI-powered self-care applications [21]. Therefore, there is a need for patient education to equip them with effective knowledge and skills to utilize AI-powered applications in self-care for OHCA. At-risk populations from low-income families lack access to AI-powered self-care tools and digital devices.

Limitations and Recommendations for Future Studies

This review was specifically interested in the use of AI in OHCA, with strict inclusion criteria - only peer-reviewed, full-text articles in English from the past five years were eligible - resulting in a small pool of just 10 studies and reduced external validity. In this regard, it is recommended that newer randomized controlled trials (RCTs) be conducted to improve the validity and reliability of the findings.

Publication bias is another limitation of this study. Since the study is a systematic review of existing research studies, the findings and conclusions carry forward bias in the studies that were included during the search process. The above can be addressed by using RCTs, which have minimal bias in their studies. Variability in implementing AI across the studies that were reviewed is another limitation of the current study. Authors of the reviewed studies applied AI in different contexts, which introduces variance in the findings and results. Therefore, future RCTs about the application of AI in OHCA should strive to include studies that uniformly implement AI within the context of OHCA.

## Conclusions

This study aimed to explore the role of AI in enhancing immediate response and successful management of cardiac arrest by improving the recognition of cardiac arrest from emergency calls in an out-of-hospital setting. The findings reveal that the integration of AI facilitates the early prediction of potential cases of cardiac arrest in out-of-hospital settings. DL and ML features enable emergency care providers and medical dispatchers to predict patients who are at a greater risk of cardiac arrest than when standard care only is utilized. Early prediction is associated with improved decision-making regarding the next action steps to take. Besides, AI enhances self-care, whereby through virtual doctors and online applications, patients can take proactive measures when they are susceptible to a heart attack by using AI-powered applications like the atrial fibrillation app. A proactive action ensures a timely response, which boosts patient outcomes. As a result, the integration of AI significantly improves patient outcomes. The application of AI in OHCA self-care, in particular, comes with negative impacts on data privacy and accountability. For instance, patient data will be vulnerable to unauthorized access, and care providers will not be held accountable for potentially harmful decisions that patients make on their own.

These findings have policy implications as follows. First, governments should fund initiatives aimed at integrating AI applications in OHCA to boost access to digital devices and AI-powered tools among consumers. Second, a policy mandating healthcare organizations to train care providers and patients on the effective application of AI in OHCA to address digital illiteracy challenges and boost self-care and self-management outcomes. However, implementing these recommendations faces key challenges: high financial costs, time and resource demands for training, and resistance to adopting AI in OHCA.
